# A phase 2 study of abemaciclib in patients with brain metastases secondary to non-small cell lung cancer or melanoma

**DOI:** 10.1093/noajnl/vdag005

**Published:** 2026-01-18

**Authors:** Solmaz Sahebjam, Danielle A Bazer, Emilie Le Rhun, Paola Queirolo, Guy Jerusalem, Erica L Johnston, Pierfranco Conte

**Affiliations:** Department of Oncology, The Sidney Kimmel Comprehensive Cancer Center at Johns Hopkins, Johns Hopkins University School of Medicine,Baltimore, Maryland, USA (S.S., D.A.B.); Department of Oncology, The Sidney Kimmel Comprehensive Cancer Center at Johns Hopkins, Johns Hopkins University School of Medicine,Baltimore, Maryland, USA (S.S., D.A.B.); Cooper Neurological Institute, Cooper University, Camden, New Jersy, USA (D.A.B.); Department of Medical Oncology and Hematology, Zurich University Hospital, Zurich, Switzerland (E.L.R.); Division of Medical Oncology for Melanoma, Sarcoma, and Rare Tumors, IRCCS Ospedale Policlinico San Martino, Genova, Italy (P.Q.); Medical Oncology Department, Centre Hospital Universitaire, Liege, Belgium (G.J.); Eli Lilly and Company, Indianapolis, IN, USA (E.L.J.); Division of Medical Oncology, University of Padova and Instituto Oncologico Veneto, Padova, Italy

**Keywords:** abemaciclib, brain metastasis, phase II clinical trial

## Abstract

**Background:**

Abemaciclib is a selective cyclin-dependent kinase 4 and 6 inhibitor that penetrates the blood-brain barrier, resulting in comparable concentrations in tissue and plasma. The primary objective of this nonrandomized Simon two-stage phase II trial (NCT02308020) was to evaluate the intracranial objective response rate in patients with brain metastases secondary to breast cancer (already published), non-small cell lung cancer (NSCLC), or ­melanoma receiving abemaciclib. Secondary objectives evaluated safety, extracranial response, progression-free survival (PFS), and overall survival (OS).

**Methods:**

Eligible subjects were enrolled in NSCLC or melanoma tumor-specific cohorts and treated with abemaciclib 200 mg twice daily (BID) monotherapy or 150 mg BID for NSCLC patients on concurrent pemetrexed or gemcitabine.

**Results:**

A total of 51 patients were enrolled (NSCLC, *n = *28; melanoma, *n = *23). No confirmed intracranial response was observed in either cohort. Volumetric decrease in target intracranial lesions was 22.7% for NSCLC and 18.8% for melanoma cohorts. Intracranial clinical benefit rate was 26.1% (95% CI: 8.1-44) for NSCLC and 9.1% (95% CI: 0-21.1) for melanoma cohorts. In the NSCLC cohort, median OS and PFS were 7.1 months (95% CI: 3.7-9.4) and 1.6 months (95% CI: 1.4-3.5), respectively. In the melanoma cohort, median OS and PFS were 2.9 months (95% CI: 1.2-4.3) and 1.4 months (95% CI: 1.0-2.0), respectively. Abemaciclib safety was consistent with previously reported data.

**Conclusions:**

Although abemaciclib can achieve therapeutic concentrations in brain metastases tissue, this study did not meet its primary endpoint. The limited clinical activity in this study suggests that further clinical trials should focus on the use of abemaciclib combination therapy.

Key Points

- Abemaciclib can achieve therapeutic concentration in brain metastases tissue.
- The limited clinical activity observed in this study suggests that further clinical trials should focus on use of abemaciclib in combination with agents with synergistic mechanisms, rather than monotherapy.

Importance of Study

Although preclinical data for abemaciclib were promising, this study did not reach its primary endpoint. The surgical cohort confirmed that abemaciclib and its metabolites cross the blood-brain barrier and reach unbound levels expected to produce cyclin-dependent kinase (CDK) 4 and CDK6 inhibition and cell-cycle arrest. Consistent with previously reported data, this suggests that abemaciclib has strong central nervous system pharmacokinetics.

Introduction

Brain metastases occur in a significant number of adults with cancer, with the highest incidence in lung cancer (40%-50%), followed by breast cancer (15%-25%), and melanoma (5%-20%).^1^ With the concurrent improvement of systemic therapies leading to prolonged survival and earlier detection of brain metastases, more intracranial recurrences are observed.^2^ Although targeted anticancer therapies have shown promising results in treating extracranial disease, the ability for these agents to cross the blood–brain barrier and efficiently deliver therapeutic effects to brain metastases remains a challenge.

Abemaciclib is an oral, selective, and potent small molecule inhibitor of cyclin-dependent kinases (CDK) 4 and 6. In vivo target inhibition studies have shown that abemaciclib inhibits retinoblastoma protein (Rb) phosphorylation, leading to a G1 arrest and inhibition of cell proliferation.^3^ Abemaciclib has demonstrated efficacy out to 5 years and is indicated in combination with endocrine therapy (tamoxifen or an aromatase inhibitor) for the adjuvant treatment of adult patients with hormone receptor (HR)-positive, human epidermal growth factor receptor 2 (HER2)-negative, node-positive, early breast cancer at high risk of recurrence.^4^^,^^5^ Abemaciclib in combination with endocrine therapy is globally approved as either upfront treatment or following progression on or after endocrine therapy for adult patients with HR+, HER2− advanced breast cancer.^6–8^ In addition, abemaciclib monotherapy is approved for adult patients with heavily pretreated HR+, HER2− metastatic breast cancer, following progression on chemotherapy for advanced disease.^9^ In the first in human study, single-agent abemaciclib demonstrated tolerability and antitumoral effects with radiographic responses in patients with heavily pretreated NSCLC and melanoma.^10^ In preclinical studies, Raub et al. demonstrated that abemaciclib crosses the blood-brain barrier and prolongs survival in orthotopic U87MG intracranial glioblastoma xenograft models.^11^ Based on these clinical and pre-clinical data, this phase 2 non-randomized multicohort trial (NCT02308020) was designed to evaluate intracranial and extracranial activity of abemaciclib in patients with brain metastases secondary to HR+ breast cancer, NSCLC, and melanoma. In addition, a surgical cohort was included to study blood–brain barrier penetrance of abemaciclib by measuring concentrations of abemaciclib and its active metabolites in plasma, cerebrospinal fluid, and brain metastases tissue after receiving abemaciclib for 5-14 days prior to intracranial surgical resection.^12^  The results of the HR+ breast cancer arm and the surgical cohort have been reported previously.^12^ In this manuscript, we will report the results of the NSCLC and melanoma cohorts.

## Methods

### Study Design

**Figure 1.**
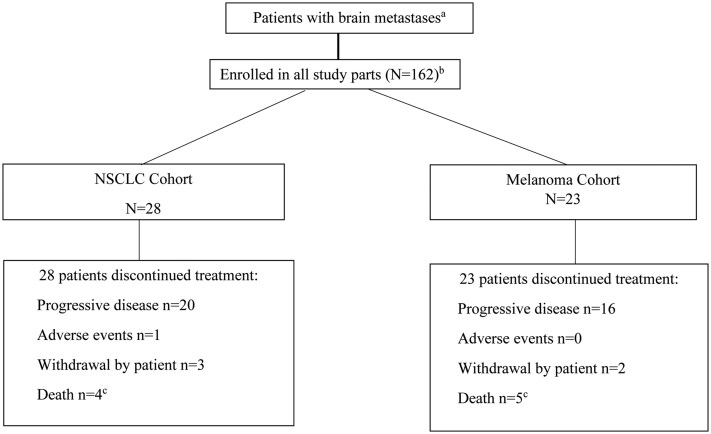
Study design and patient disposition.^a^ Patients not randomized to treatment. All patients who received at least 1 dose of abemaciclib.^b^ Additional cohorts enrolled a total of 112 patients with brain metastases secondary to HR+ breast cancer; this data has been published.^12^  ^c^In the NSCLC cohort, two deaths were due to AEs and 2 due to study disease. In the Melanoma cohort, all five deaths were due to the study disease.

This was an open-label, non-randomized, multicenter Simon two-stage II trial of abemaciclib in which patients with brain metastases secondary to HR+ breast cancer, NSCLC, or melanoma were enrolled into tumor-specific cohorts (Figure 1).   This trial was registered in ClinicalTrials.gov (NCT02308020), and the study protocol was approved by institutional review boards and ethics committees. The study was conducted in accordance with the Declaration of Helsinki. All patients provided written informed consent prior to study participation and enrollment.

### Eligibility Criteria

Patients were eligible if they had either (1) ≥1 new or not previously irradiated measurable metastatic brain lesion ≥ 10 mm in the longest diameter and ≥5 mm in the perpendicular plane according to Response Assessment in Neuro-Oncology Criteria for Brain Metastases (RANO-BM)^13^ or (2) a progressive previously irradiated brain metastasis secondary to NSCLC or melanoma by local assessment; had completed local therapy (surgical resection or stereotactic radiosurgery) ≥ 14 days prior to initiating abemaciclib and recovered from all acute effects; on a stable or decreasing dose of concomitant corticosteroids (if clinically indicated) for at least 7 days prior to the baseline gadolinium-enhanced magnetic resonance imaging (MRI); had a Karnofsky performance status of ≥ 70, and life expectancy ≥ 12 weeks. Prior treatment with any CDK4/6 inhibitor was not allowed, and evidence of leptomeningeal disease was excluded. NSCLC patients receiving gemcitabine or pemetrexed at enrollment were permitted to continue treatment with either (per investigator discretion) as long as there was evidence of CNS progression but stable extracranial disease for ≥ 6 weeks prior to study entry.

### Treatment

Abemaciclib 200 mg monotherapy was administered orally twice daily on a continuous 21-day cycle. For patients with NSCLC brain metastases receiving concurrent treatment with gemcitabine or pemetrexed, abemaciclib 150 mg twice daily was administered. All patients remained on study treatment until disease progression, unacceptable toxicity, or other discontinuation criteria were met.

### Efficacy and Safety Assessments

Intracranial and extracranial tumor assessments were performed at baseline (obtained less than 28 days before commencement of treatment), every 6 weeks for the first 24 weeks, and then every 12 weeks thereafter until progressive disease. Progression of intracranial target (maximum of five) and non-target lesions was assessed using contrast-enhanced MRI and according to RANO-BM criteria. Extracranial responses were measured using computed tomography (CT) scans or MRI and assessed per RECISTv1.1.^14^

Physical exams and laboratory assessments were performed prior to the start of each cycle. Treatment-emergent adverse events (TEAEs) were assessed and graded according to the National Cancer Institute Common Terminology Criteria for Adverse Events (CTCAE) v4.0.^15^

### Study Endpoints

The primary endpoint was investigator-assessed objective intracranial objective response rate (OIRR; confirmed complete response [CR] + partial response [PR]) in accordance with RANO-BM criteria in the OIRR evaluable population.

Secondary endpoints used the full analysis set (FAS) population. Secondary intracranial endpoints included best overall intracranial response (iBOR), duration of intracranial response (CR + PR), intracranial disease control rate (iDCR; CR + PR + stable disease), and intracranial clinical benefit rate (iCBR; CR + PR + Stable disease ≥ 6 months). Secondary extracranial endpoints included objective response rate (eORR; CR + PR), disease control rate (eDCR; CR + PR + stable disease), and clinical benefit rate (eCBR; CR + PR + stable disease ≥ 6 months) per RECIST version 1.1.

In addition, median intracranial, extracranial, and bicompartmental progression-free survival (PFS) were assessed. Bicompartmental PFS was measured from the enrollment date to the earliest date of either intracranial or extracranial disease progression, or death from any cause. Overall survival (OS) was calculated from enrollment date to date of death from any cause.

### Statistical Analysis

Simon two-stage design was used for interim analysis with OIRR as the decision criterion to open the second stage, assuming a 0.05 one-sided type-I error and 80% power. The null hypothesis of true abemaciclib OIRR ≤5% versus ≥15% was tested per disease cohort. If stage 1 had ≥2 responders of 23 patients, accrual continued to the second stage until 33 additional patients were enrolled. Six responders out of 56 enrolled patients were required to warrant further investigation of abemaciclib in these patient populations.

All analyses included patients who received at least 1 dose of abemaciclib. All time-to-event outcomes were analyzed by Kaplan–Meier estimates and reported with two-sided 95% confidence intervals (CI). Analyses were conducted with SAS version 9.2 (SAS Institute Inc.).

## Results

### Patient Characteristics and Treatment

Between April 2015 and October 2018, 51 patients (28 patients in the NSCLC cohort; 23 patients in the melanoma cohort) were enrolled and received ≥1 dose of abemaciclib (Figure 1). At time of study entry, the majority of patients were symptomatic with a KPS ≤ 80%, had received ≥ 2 prior systemic therapies in the metastatic setting, and the median no. of intracranial target lesions was 1.0 (1.0-5.0) in the NSCLC cohort and 2.0 (1.0-5.0) in the melanoma cohort. Most patients had prior radiation therapy to the brain (20 [71.4%] patients with NSCLC and 16 [69.6%] patients with melanoma): whole brain radiotherapy (NSCLC: 39.3%; melanoma: 43.5%) and stereotactic radiosurgery (NSCLC: 32.1%; melanoma: 26.1). The median time from radiation to study start in NSCLC and melanoma cohorts was 4.8 and 5.6 months, respectively. In the NSCLC cohort, three patients received combination pemetrexed, and no patients continued gemcitabine on study. Patient demographics, baseline disease characteristics, and prior therapies are delineated in Table 1.

**Table 1.** Baseline patient and disease characteristics

	NSCLC	Melanoma
*n* (%), unless otherwise stated	*n* = 28	*n* = 23
Age, years median (range)	59.5 (34-79)	55 (32-77)
Female	14 (50.0)	11 (47.8)
Male	14 (50.0)	12 (52.2)
Race, White	20 (71.4)	22 (95.7)
KPS		
≥90	12 (42.9)	9 (39.1)
80	12 (42.9)	7 (30.4)
70	4 (14.3)	6 (26.1)
Prior systemic therapy		
1	12 (42.9)	7 (30.4)
2	7 (25.0)	7 (30.4)
3	3 (10.7)	5 (21.7)
4 or more	4 (14.3)	4 (17.4)
No. of target intracranial lesions (median)	1.0 (1.0-5.0)	2.0 (1.0-5.0)
Prior treatments of target intracranial lesions:		
WBRT	11 (39.3)	10 (43.5)
SRS	9 (32.1)	6 (26.1)
Surgical resection	4 (14.3)	2 (8.7)
Time from radiation to treatment start, months, median	4.8	5.6
*KRAS* mutation	10 (37.0)	0
*G12C*	4 (40.0)	
*G12D*	1 (10.0)	
*G12V*	1 (10.0)	
*BRAF* mutation	1 (4.0)	13 (56.5)
*BRAFV600E mutation*	0	10 (76.9)
*EGFR* mutation	1 (3.7%)	0

**Table 2.** Summary of adverse events

	NSCLC		Melanoma	
	(*n* = 28)		(*n* = 23)	
Total patients with ≥ 1 TEAE	27 (96.4)		18 (78.3)	
	TEAE of any Grade, *n* (%)	TEAE Grade 3/4, *n* (%)	TEAE of any Grade, *n* (%)	TEAE Grade 3/4, *n* (%)
Diarrhea	15 (53.6)	0	5 (21.7)	0
Fatigue	11 (39.3)	3 (10.7)	4 (17.4)	1 (4.3)
Nausea	8 (28.6)	0	1 (4.3)	0
Thrombocytopenia	8 (28.6)	2 (7.1)	3 (13.0)	0
Neutropenia	7 (25.0)	5 (17.9)	5 (21.7)	0
Anemia	5 (17.9)	1 (3.6)	2 (8.7)	1 (4.3)
Vomiting	4 (14.3)	0	1 (4.3)	0
Headache	4 (14.3)	0	2 (8.7)	0
Hypokalemia	2 (7.1)	1 (3.6)	1 (4.3)	0
Dyspnea	6 (21.4)	2 (7.1)	0	0
Abdominal pain	2 (7.1)	0	5 (21.7)	0
Leukopenia	4 (14.3)	3 (10.7)	3 (13.0)	0
Lymphopenia	4 (14.3)	1 (3.6)	1 (4.3)	0

Abbreviations: TEAE, treatment emergent adverse event.

### Intra- and Extracranial Activity

#### NSCLC Cohort

**Figure 2.**
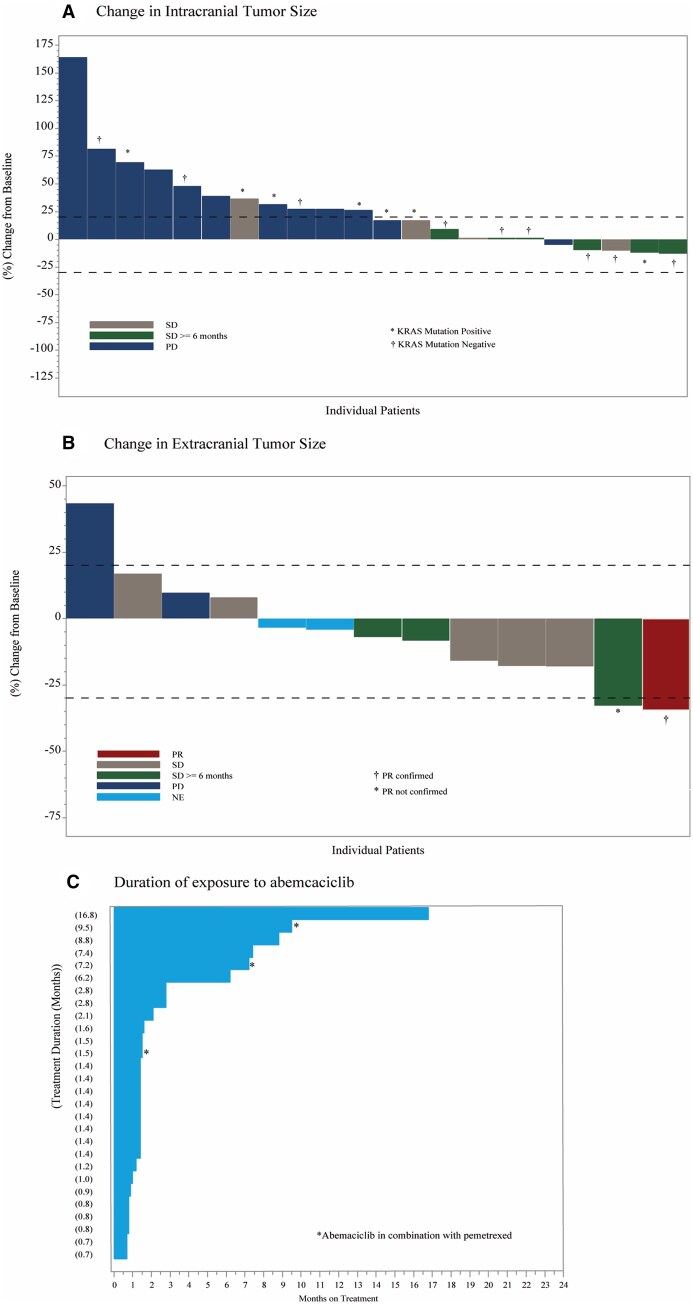
Change in intracranial (A) and extracranial (B) tumor size and best overall response, and duration of exposure to abemaciclib (C) in the NSCLC cohort. Abbreviations: NE = Not Estimable; PR = Partial Response; PD = Progressive Disease; SD = Stable Disease.

At the time of data cut-off on November 8, 2018, all NSCLC patients (*n* = 28) had discontinued abemaciclib. Progressive disease was the most common reason for study treatment discontinuation, occurring in 20 (71.4%) patients; other reasons included 4 (14.3%) deaths, 3 (10.7%) withdrawal by subject, and 1 (3.6%) due to adverse events. In the OIRR evaluable population, a decrease in the size of intracranial target lesions was observed in 5 (22.7%) patients (Figure 2). However, they did not qualify as a response per RANO-BM criteria resulting in confirmed OIRR of 0% and closure of this cohort at the end of stage I. In the FAS population, a best response of iSD occurred in 10 patients, resulting in iDCR of 35.7% (95% CI, 18.0%-54%). The iSD lasted ≥ 6 months in six patients, for an iCBR of 21.4% (95% CI, 6.2-36.6). With respect to extracranial disease, 1 confirmed PR was observed (Figure 2), for an ORR of 4.0% (95% CI, 0.0-11.7).

In the FAS population, the median iPFS was 1.55 months (95% CI, 1.35-3.52) with 16 (57.1%) PD events, and the median bicompartmental PFS was 1.45 months (95% CI, 1.35-2.76). Numerical difference compared to iPFS was observed in extracranial PFS, with 4.9 months (95% CI, 2.66-7.50). Median OS was 7.13 months (95% CI, 3.65-9.37) with 24 deaths (85.7%) at time of analysis, and 9-month OS rate of 38.9% (95% CI, 20.7-56.7).

#### Melanoma Cohort

**Figure 3.**
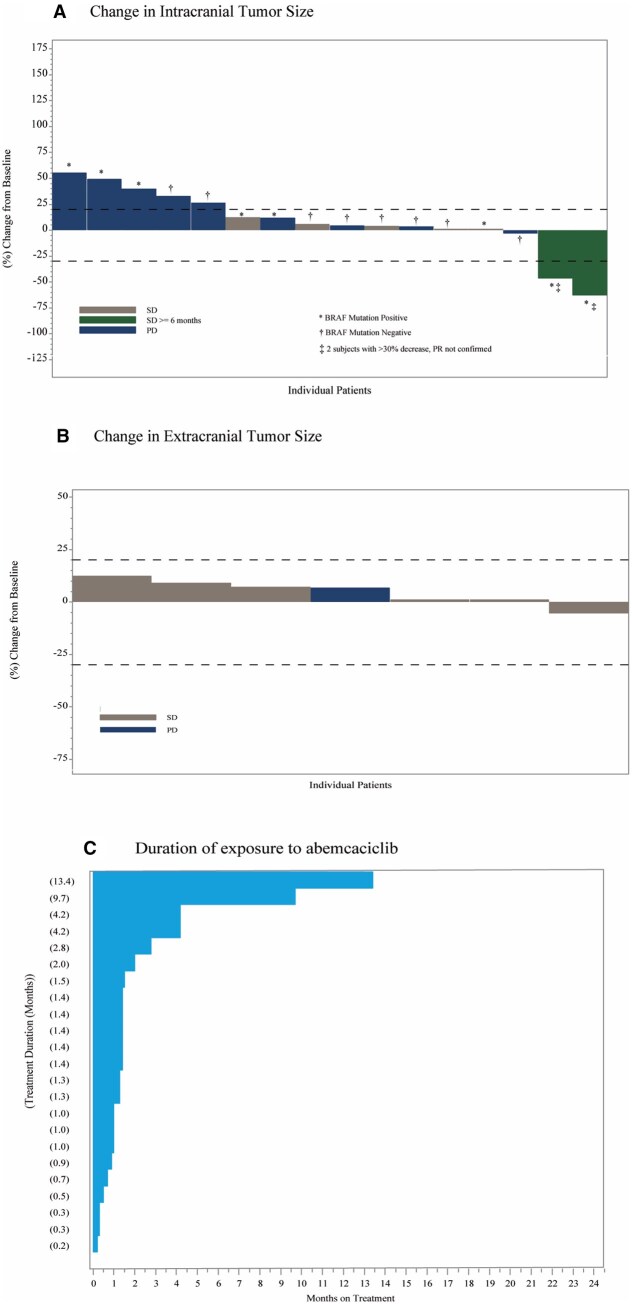
Change in intracranial (A) and extracranial (B) tumor size and best overall response, and duration of exposure to abemaciclib (C) in Melanoma cohort. Abbreviations: PD= Progressive Disease, SD = Stable Disease.

At data cut-off, all patients (*n* = 23) had discontinued abemaciclib. Reasons for treatment discontinuation were disease progression (*n *= 16, 69.6%), death (*n *= 5, 21.7%), and withdrawal (*n* = 2, 8.7%). In the OIRR evaluable population, a decrease in the size of intracranial target lesions was observed in three (18.8%) patients (Figure 3). However, no confirmed intracranial responses were observed, and this cohort was also closed at the end of stage I. In the FAS population, best response of iSD occurred in seven patients, resulting in iDCR of 30.4% (95% CI, 11.6-49.2). Two patients had an iSD of ≥ 6 months, for an iCBR of 8.7% (95% CI, 0.0-20.2). No extracranial responses were observed.

Intracranial progressive disease events occurred in 14 (60.9%) patients. The median iPFS and bicompartmental PFS in the FAS population were 1.38 months (95% CI, 1.02-1.97) and 1.22 months (95% CI, 1.02-1.55), respectively. Median extracranial PFS was similar (1.41 months; 95% CI, 1.15-4.08). Median OS was 2.93 months (95% CI, 1.22-4.31), with 19 deaths (82.6%) at time of analysis, and a 6-month OS rate of 30.1% (95% CI, 12.5-50.0).

### Safety and Tolerability

The median number of cycles received for both NSCLC and melanoma cohorts was two cycles. Observed TEAEs were consistent with previously reported adverse reactions associated with abemaciclib. No new safety signals were observed. In the NSCLC cohort, the most common any grade TEAEs (≥ 20%) were diarrhea (53.6%), fatigue (39.3%), nausea (28.6%), thrombocytopenia (28.6%), and neutropenia (25.0%). Grade ≥ 3 TEAEs occurring in ≥10% of patients included neutropenia (17.9%), fatigue (10.7%), and leukopenia (10.7%).

In the melanoma cohort, the most common TEAEs of any grade (≥ 20%) were diarrhea (21.7%), neutropenia (21.7%), and abdominal pain (21.7%). Fatigue occurred in 17.4% of patients. No grade ≥ 3 AEs were reported in ≥10% of the patients.

Dose adjustments (at least 1 dose omission and/or reduction) were required in 18 (64.3%) patients in the NSCLC cohort and 5 (21.7%) patients in the melanoma cohort. Of these 18 NSCLC patients requiring dose adjustment, 8 (28.6%) patients required a dose reduction due to adverse event, of which only 2 (7.1%) patients required two dose reductions, and only 1 (3.6%) patient discontinued due to AE. Of the five melanoma patients, only 1 (4.3%) patient required a single dose reduction, and no patients discontinued due to AE.

Sixteen deaths (31.4%) occurred while on treatment or within 30 days of treatment discontinuation, *n* = 6 in the NSCLC cohort (21.4%) and *n* = 10 patients in the melanoma cohort (43.5%), the majority (81.3%) were secondary to underlying malignancy. Three deaths in the NSCLC cohort were due to AEs not related to treatment; no deaths due to AEs occurred in the melanoma cohort.

## Discussion

Lack of drug penetration across the blood–brain barrier to achieve therapeutic concentrations, and primary or acquired drug resistance are the two major reasons for the failure of clinical trials investigating the efficacy of novel anti-cancer agents in patients with either primary brain tumors or brain metastases. Although the rationale based on preclinical studies of CDK4/6 inhibitors and early-phase clinical trials in lung cancer and melanoma was promising, our study investigating the role of abemaciclib in the treatment of brain metastases secondary to NSCLC or melanoma did not meet its primary endpoint.

In this study and previously reported data, the surgical cohort confirmed that abemaciclib and its metabolites cross the blood–brain barrier and reach unbound levels expected to produce CDK4 and CDK6 inhibition and cell-cycle arrest. In patients from the surgical cohort, unbound brain metastases concentrations of total active abemaciclib analytes were 96- [cyclin dependent kinase 4 (CDK4)] and 19-fold (CDK6) above in vitro IC50. Furthermore, steady-state plasma and CSF exposures were consistent with those associated with reductions in RB protein and DNA topoisomerase II alpha G1 arrest in xenograft models (7). Hence, failure to achieve a therapeutic concentration in brain metastases is unlikely to be the etiology of this study’s lack of response in NSCLC and melanoma cohorts. While the sample size was small to conduct comparative analyses, the late line of therapy and symptomatic and/or high disease burden in these cohorts may likely be confounding reasons we did not observe objective intracranial responses in these patient populations.

In preclinical studies, synthetic lethal interaction has been observed between *KRAS* oncogenes and CDK4 in a genetically engineered mouse model of NSCLC.^16^ Furthermore, pharmacologic experimentation of six human xenograft models of NSCLC showed that tumors with the *KRAS* mutation were more sensitive to abemaciclib than *KRAS* wild-type tumors.^10^ Consistent with pre-clinical data, in the phase I study of abemaciclib, patients with advanced NSCLC harboring the *KRAS* mutation had improved disease control rates when compared to those with *KRAS* wild-type tumors. However, in this study’s NSCLC cohort, we had 10 patients with *KRAS* mutant tumors (10/28). Yet, no intracranial response was observed, and the PFS and OS were comparable between patients with *KRAS* wildtype and mutant tumors. These data are in line with the previously reported phase 3 trial investigating abemaciclib versus erlotinib in patients with stage IV systemic NSCLC with the *KRAS* mutation, which failed to show a significant OS benefit with the use of abemaciclib.^17^ Numerically, no notable difference in response was observed for patients who had received prior intracranial radiation therapy vs not. In the NSCLC cohort, three of six patients with intracranial SD ≥ 6 months had targeted SRS, of which two also received WBR, while in the melanoma cohort, only one of three patients with intracranial SD ≥6 months received targeted SRS. Given the brain penetration and exposure concentration achieved with abemaciclib in this study, combining abemaciclib with a novel agent targeting KRAS G12C or G12D mutations may be of interest and provide therapeutic benefit for these NSCLC patients.

The abemaciclib safety data obtained in the NSCLC and melanoma cohorts were consistent with prior studies. No new safety signals, neurologic or non-neurologic, were observed in this patient population with brain metastases. Fatigue and diarrhea were the most common adverse events. Diarrhea is the most common side effect of abemaciclib, which was primarily low grade and can be well managed with antidiarrheal medication at the first sign of loose stool, and subsequent, as-needed, dose adjustments.^18^

Limitations should be considered when interpreting data from this study, as this is a small, nonrandomized trial without a control arm and no comparative analyses were conducted. In addition, the patient population was heterogeneously treated for the systemic disease prior to enrollment in our study and may have had inherent or acquired treatment-resistant disease, given the nature of the disease course and prior treatment history. Furthermore, the lack of translational data is a limiting factor in further selecting a subgroup of patients that may have benefited from abemaciclib.

Since the design and execution of our study, new literature has explored synergistic combinations of CDK4/6 inhibitors with immunotherapy or molecularly targeting agents.^19^^,^^20^ Abemaciclib has been shown to induce a T cell–inflamed tumor microenvironment and enhance the efficacy of PD-L1 checkpoint blockade.^19^ Moreover, MAPK/ERK inhibition in conjunction with CDK 4/6 inhibition has been tested and appears to be promising synergistically. In BRAF/MEK inhibitor–resistant patient-derived xenograft models of melanoma, the combination of CDK4/6 and MEK inhibition significantly decreased tumor growth, suggesting that the combination of CDK4/6 and MEK inhibitors may serve as a therapeutic strategy to overcome acquired resistance to BRAF/MEK blockade.^20^ Furthermore, novel agents such as SHP2 inhibitors are emerging as potential partners for combination therapy.^21^^,^^22^ As anti-PD1 antibodies and dual BRAF/MEK inhibition are considered mainstay treatment options for advanced NSCLC and melanoma, these combinations warrant further exploration in patients with brain metastases from NSCLC or melanoma. Real world study^23^ and ongoing non-sponsored trials in patients with brain metastases or primary CNS tumors are assessing abemaciclib monotherapy (NCT05413304, NCT02523014, NCT05940493) or in combination with elacestrant (NCT05386108), and with SRS plus endocrine therapy (NCT04923542). In summary, our data, combined with results from other clinical trials suggest that CDK4/6 inhibitors, including abemaciclib, do not show benefit as a single agent for treating brain metastases secondary to NSCLC or melanoma. Given the strong central nervous system pharmacokinetics of abemaciclib and emerging evidence indicating synergistic potential of CDK4/6 inhibitors, further evaluations of abemaciclib-based combinations are warranted in patients with brain metastases or primary CNS tumors.

## Data Availability

Data available on request. Lilly provides access to all individual participant data collected during the trial, after anonymization, with the exception of pharmacokinetic or genetic data. Data are available to request 6 months after primary publication acceptance. No expiration date of data requests is currently set once data are made available. Access is provided after a proposal has been approved by an independent review committee identified for this purpose and after receipt of a signed data sharing agreement. Data and documents, including the study protocol, statistical analysis plan, clinical study report, blank or annotated case report forms, will be provided in a secure data sharing environment. For details on submitting a request, see the instructions provided at www.vivli.org.
